# Comparison of Antidiabetic Medications during the Treatment of Atherosclerosis in T2DM Patients

**DOI:** 10.1155/2017/5032708

**Published:** 2017-04-28

**Authors:** Xiaojie Liu, Tao Mei, Wei Chen, Shandong Ye

**Affiliations:** ^1^School of Medicine, Shandong University, Jinan, Shandong, China; ^2^Department of Geriatrics, Anhui Provincial Hospital Affiliated to Anhui Medical University, Hefei, China; ^3^Department of MEC, Anhui Provincial Hospital Affiliated to Anhui Medical University, Hefei, China; ^4^Department of Nephrology, Anhui Provincial Hospital Affiliated to Anhui Medical University, Hefei, China; ^5^Department of Endocrinology, Anhui Provincial Hospital Affiliated to Anhui Medical University, Hefei, China

## Abstract

Type 2 diabetes is often associated with arterial atherosclerosis in large blood vessels. We set out to elucidate whether commonly used antidiabetic drugs metformin, sitagliptin, and pioglitazone will reduce atherosclerosis in T2DM patients. We enrolled 176 individuals with type 2 diabetes, which were divided into four treatment groups according to different oral drugs: metformin alone, sitagliptin alone, pioglitazone alone, or combination of metformin and sitagliptin. We assessed changes in glycometabolism, lipid metabolism, cytokine released, and carotid artery intima-media thickness as the readout for improvement in atherosclerosis. HbA1c levels were significantly decreased in all treatment groups (*p* < 0.05), and FBG levels were also decreased in metformin and combined groups (*p* < 0.05). In addition, we found IL-6 levels significantly decreased in all treatment groups (*p* < 0.05). Treatment with pioglitazone showed a significant increase in BMI, HDL, and ADPN levels (*p* < 0.05). We also observed a significant decrease in NHDL levels in the combined treatment group (*p* < 0.05). Our data revealed that in addition to hypoglycemic properties of metformin, sitagliptin, and pioglitazone, these drugs also have the potential to promote an anti-inflammatory response. Therefore, combination therapy may be more beneficial for reducing atherosclerosis in patients with type 2 diabetes. The clinical trial is registered with ChiCTR-ORC-17010835.

## 1. Introduction

Atherosclerosis is one of the most severe complications associated with Type 2 diabetes mellitus (T2DM), often progressing to myocardial infarction and cerebral stroke. Accordingly, atherosclerosis is an important cause of disability and mortality in T2DM patients [1]. Risk factors that contribute to the development of atherosclerosis include hyperglycemia, increased low density lipoprotein-cholesterol (LDL-C), and elevated triglyceride levels [2]. Previously, Ross et al. reported that an inflammatory response occurs during the development of atherosclerosis [3].

Inflammation associated with T2DM is performed mainly by the release of proinflammatory cytokines, such as Interleukin-6 (IL-6) and tumor necrosis factor-alpha (TNF-*α*), by multiple tissues. Meanwhile, the secretion of antiinsulin resistance cytokines, such as adiponectin (ADPN) and IL-10, is reduced [4]. Proinflammatory cytokines act upon vascular endothelial cells (VECs) leading to the reduction of nitric oxide (NO) production, followed by VEC damage [5]. In addition, these cytokines attract and promote the attachment of mononuclear cells, granular cells, and T cells to VECs, which lead to artery wall inflammation, followed by atherosclerosis [4, 5]. IL-6, which is produced by activated leucocytes, adipocytes, and VECs, is a crucial proinflammatory cytokine and can affect insulin sensitivity through multiple mechanisms. IL-6 level positively correlates with the degree of insulin resistance [6]. On the contrary, ADPN is secreted by adipose tissue and works as an insulin sensitive hormone. ADPN promotes fatty acid oxidation in hepatocytes and myocytes, suppresses the production of inflammatory cytokines, protects VEC function, and reduces atherosclerosis [7].

The carotid artery is the most frequently affected blood vessel by atherosclerosis and also can serve as a window for the measurement. Early symptoms of atherosclerosis include increased thickness of intimal layer of the carotid artery. Measurement of carotid artery intima-media thickness (CCA-IMT) with ultrasonography is a safe approach for identifying and quantifying atherosclerosis in early clinical diagnosis.

The pathogenesis of T2DM is caused by damage to pancreatic islet *β*-cells and insulin resistance [8]. Metformin reduces hepatic glucose and increases insulin sensitivity of peripheral tissue thereby reducing insulin resistance. It can act on the AMP-activated protein kinase (AMPK) to modulate multiple energy pathways and promote lipid metabolism, which in turn alleviates atherosclerosis [9]. The DPP-4 inhibitor sitaglipin increases the secretion of insulin by enhancing the activities of glucagon-like peptide-1(GLP-1) and glucose-dependent insulinotropic polypeptide (GIP), delaying gastric emptying, and protecting islet *β*-cell function [10]. Previous studies have shown that sitagliptin decreases the production of proinflammatory cytokines, increases the biological availability of NO, and protects VECs, thus reducing the formation of atherosclerotic plaques [10, 11]. Pioglitazone is a highly selective PPAR*γ* agonist. It can reduce the levels of fasting insulin, hemoglobin A1c (HbA1c), and the homeostasis model of assessment for insulin resistance index (HOMA-IR). In addition, key transcription factors involved in the inflammatory response, such as nuclear factor-kappa B (NF-*κ*B) and activator protein-1 (AP-1), are suppressed by pioglitazone [12]. The purpose of this study is to investigate whether these antidiabetic drugs reduce atherosclerosis by measuring the activities of glycometabolism and lipid metabolism, release of inflammatory cytokines, and CCA-IMT.

## 2. Materials and Methods

### 2.1. Patients

We enrolled 176 T2DM patients (160 male and 16 female) at Anhui Provincial Hospital Medical Center (Hefei, China) during the period of January 2015 and November. All of the enrolled T2DM patients met the WHO-1999 T2DM diagnostic criteria. The duration of diabetes was 1–5 years. The following criteria were used for excluding patients: severe hepatic and kidney dysfunction; cardiac function grades 3-4; any systemic immune disorders; chronic inflammatory disease or tumor; severe diabetic complications; or an acute stress state within 2 months (surgical operation, active infection). Statin, ARB (angiotensin receptor blocker), and ACEI (angiotensin converting enzyme inhibitor) had not been taken. The protocol of this study was ethically approved by the Institutional Review Board of both Shandong University and Anhui Provincial Hospital in accordance with the Declaration of Helsinki.

### 2.2. Antidiabetic Medication Treatment Regimens

The 176 enrolled T2DM patients were divided into four treatment groups based on the type of antidiabetic treatment used: metformin group (*n* = 77), sitagliptin group (*n* = 31), pioglitazone group (*n* = 40), and combined treatment group (*n* = 28). For the metformin group, patients received oral administration of 1000–1500 mg of metformin daily. For the sitagliptin group, patients received oral administration of 100 mg of sitagliptin daily. For the pioglitazone group, patients received oral administration of 30 mg of pioglitazone daily. For the combined treatment group, patients received oral administration of 1000–1500 mg of metformin and 100 mg of sitagliptin daily. Dietary control and exercise options were provided.

### 2.3. Serum Cytokine ELISA

Serum was obtained from patient venous blood through centrifugation for 10 min at 1500 rpm and stored at −80°C prior to analysis. Serum IL-6 and ADPN levels were measured by enzyme-linked immunosorbent assay (ELISA) (R&D systems Minneapolis, MN, USA) according to the manufacturer's protocols.

### 2.4. Measurement of Carotid Artery Intima-Media Thickness (CCA-IMT)

The CCA-IMT value is measured at the bifurcations of the bilateral common carotid within 10 mm, in three cardiac cycles for a total for six measurements. The average of the six measurements was used to calculate the CCA-IMT. Any CCA-IMT that is below 0.9 mm was not recorded.

### 2.5. Monitoring Parameters

Patients were assessed for fasting blood glucose (FBG), hemoglobin A1c (HbA1c), triglycerides (TG), total cholesterol (TC), low density lipoproteins (LDL), high density lipoproteins (HDL), uric acid (UA), creatinine (Cr), recorded systolic pressure (SBD), measured CCA-IMT, calculated body mass index (BMI), and non-high density lipoproteins (NHDL, where NHDL = TC-HDL) at enrollment to determine baseline and subsequently reassessed following 12 months treatment. When TC level is in 2.3–5.6 mmol/L or LDL level is not high, international lipid guidelines recommend NHDL for lipid monitoring [13], especially in patients with T2DM.

### 2.6. Statistics

All statistical analysis was carried out using Graphpad and Excel using Student's *t*-test (two-tailed). Differences between multigroups were compared using one-way ANOVA.

## 3. Results

**Figure 1:**
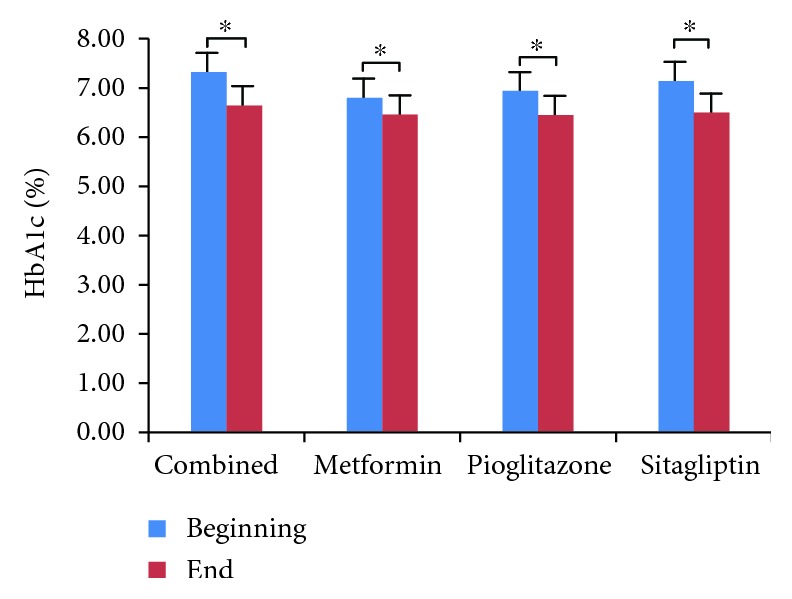
Comparison of HbA1c levels in four treatment groups. Serum HbA1c was measured by high efficiency liquid chromatography (HPLC). Data were shown as mean ± SD. HbA1c levels are significantly decreased in each treatment group (^∗^*p* < 0.05).

**Figure 2:**
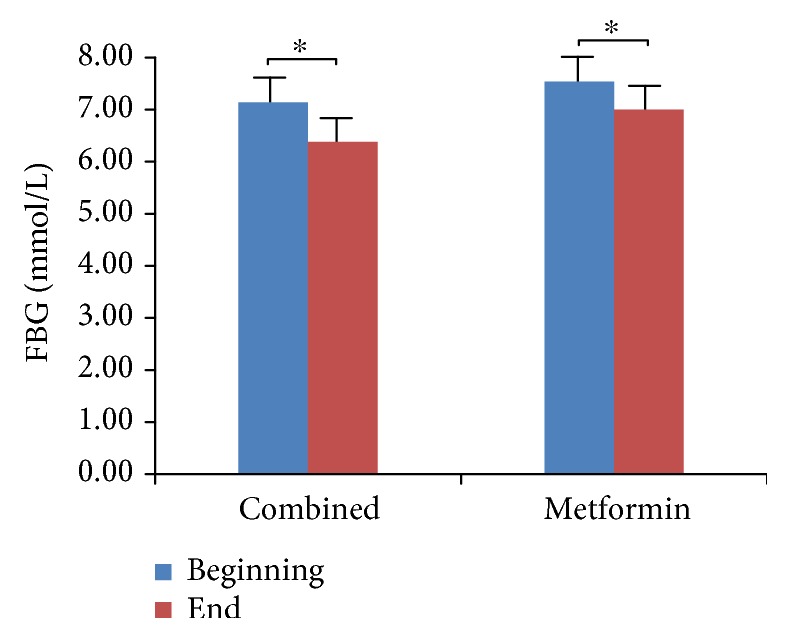
Comparison of FBG levels in metformin and combined treatment groups. Detection of FBG wanted patients with fasting 8 hours or more. Data were shown as mean ± SD. FBG levels are significantly decreased in both the metformin group and the combined treatment group (^∗^*p* < 0.05).

**Figure 3:**
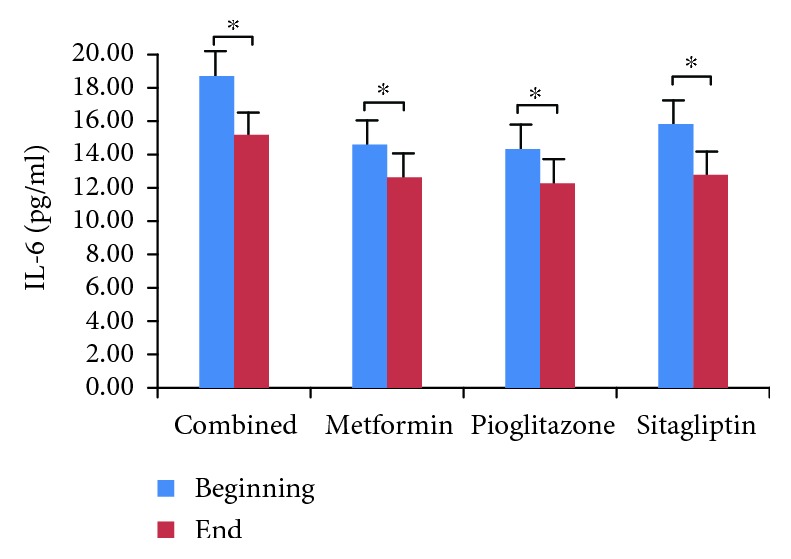
Comparison of IL-6 levels in each treatment group. Determination of serum IL-6 was by ELISA. Data were shown as mean ± SD. IL-6 levels are significantly reduced in all four groups (^∗^*p* < 0.05).

**Figure 4:**
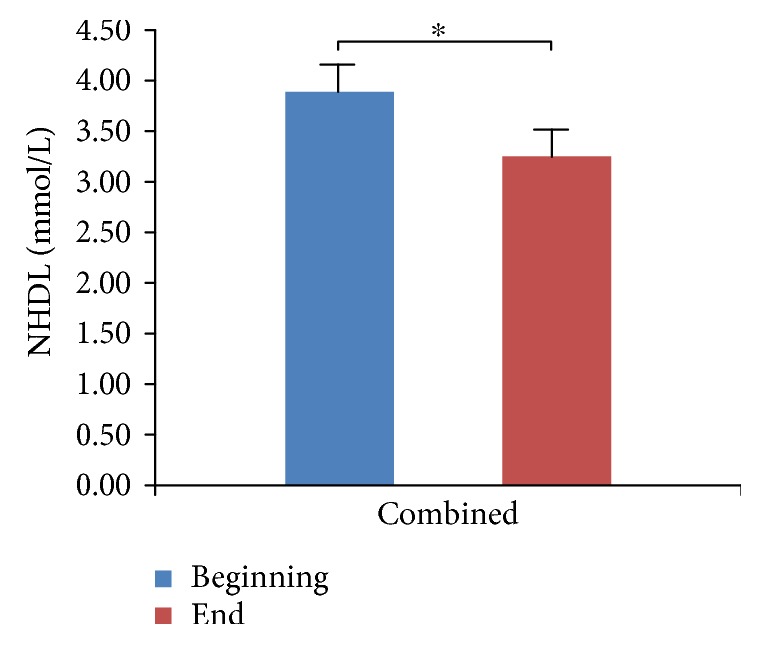
Comparison of NHDL levels in the combined treatment group. NHDL was obtained from TC-HDL. Data were shown as mean ± SD. NHDL levels are decreased in the combined treatment group (^∗^*p* < 0.05).

**Figure 5:**
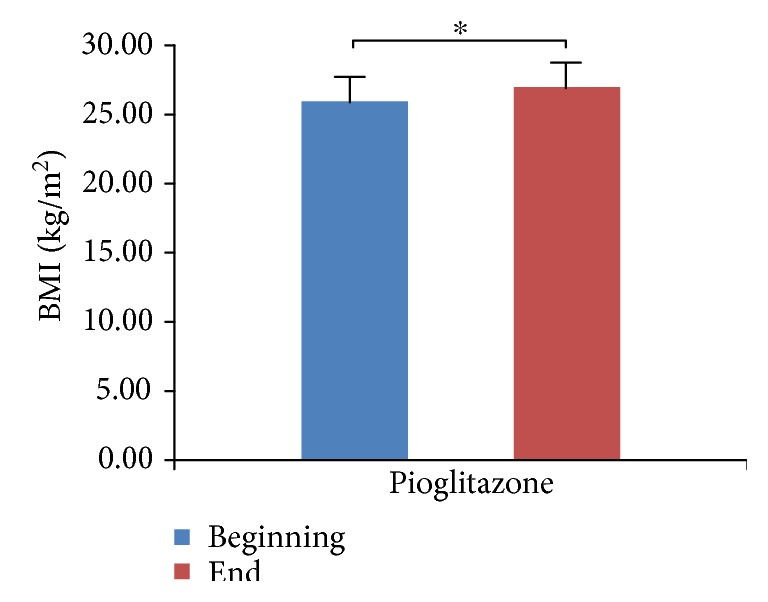
Comparison of BMI in pioglitazone treatment group. BMI = weight (kg)/height (m^2^). Data were shown as mean ± SD. BMI is significantly increased in the pioglitazone group (^∗^*p* < 0.05).

**Figure 6:**
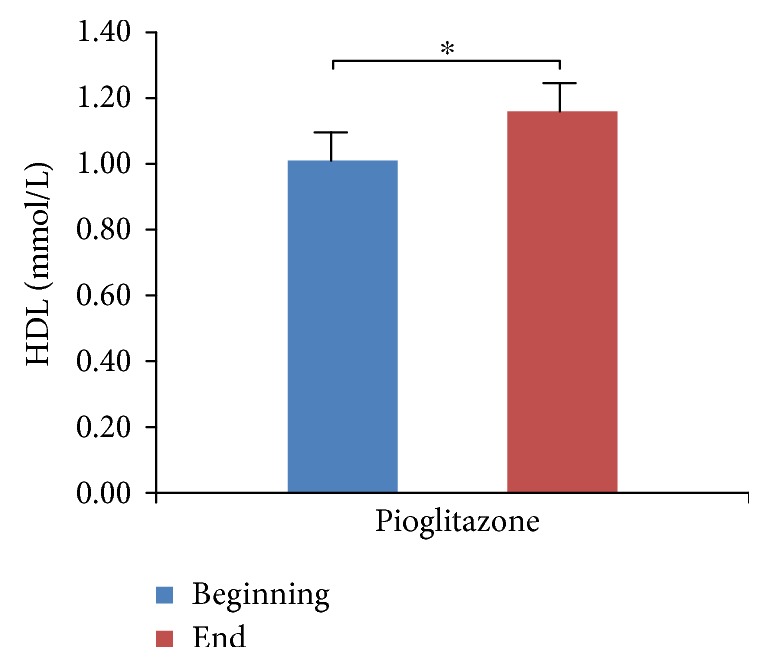
Comparison of HDL levels in the pioglitazone treatment group. Data were shown as mean ± SD. HDL levels are significantly increased in the pioglitazone group (^∗^*p* < 0.05).

**Figure 7:**
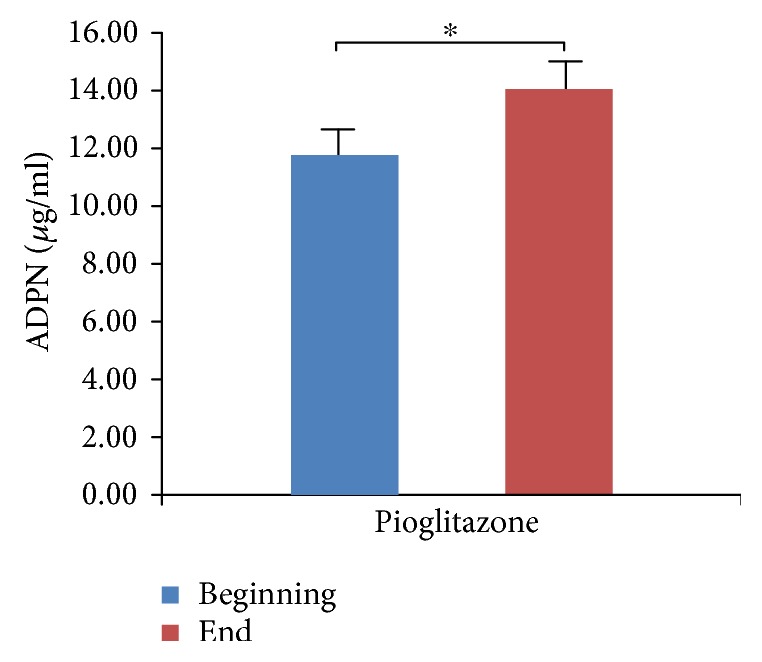
ADPN levels in the pioglitazone treatment group. Determination of serum ADPN was by ELISA. Data were shown as mean ± SD. ADPN levels are significantly increased in the pioglitazone group (^∗^*p* < 0.05).

We did not observe hypoglycemia at the endpoint of the study, although 13 patients opted out due to poor blood glucose control. We compared and analyzed the parameters of four groups (Tables 1–4). We found that HbA1c levels were significantly decreased in all four treatment groups (Figure 1, *p* < 0.05), which indicates that all three antidiabetic agents show good efficacy. FBG levels were significantly decreased in both the metformin group and the combined treatment group (Figure 2, *p* < 0.05), which suggests metformin is beneficial for T2DM that inhibited elevated FBG levels. We found IL-6 levels were significantly decreased in all four treatment groups (Figure 3, *p* < 0.05), which indicates that each of the antidiabetic drugs has anti-inflammatory property. In addition, we observed a significant decrease in NHDL levels in the combined treatment group (Figure 4, *p* < 0.05) but not in any of the single agent groups, which suggests combination treatment of metformin with sitagliptin offers additional benefits in modulating lipid metabolism. Finally, we found that BMI, HDL, and ADPN levels were significantly increased in the pioglitazone group (Figures 5–7, *p* < 0.05), which demonstrates that improved lipid metabolism and weight gain are effects of pioglitazone treatment. When IL-6 declining levels were compared in different treatment groups, no significant difference was found between each treatment group. In addition, we did not observe significant improvement of blood pressure and uric acid levels in patients.

## 4. Discussion

Our results suggest that all three antidiabetic treatments, metformin, sitagliptin, and pioglitazone, showed efficacy in reducing blood glucose levels. Metformin effectively reduced the level of HbA1C. Metformin has been considered as the first line of treatment for T2DM in many countries [14]. Although many studies have found that metformin could decrease the BMI in T2DM patients, we were unable to observe the same phenomenon in this study. This may be due to the notion that metformin-mediated weight loss is more prominent during the early stages of treatment, whereas the T2DM patients in our study had already been prescribed metformin for more than one year.

The DPP-4 inhibitor, sitagliptin, is a novel antidiabetic treatment with the ability to reduce postprandial blood glucose levels and maintain glucagon levels, improving the sensitivity of pancreatic *α*-cells to changes in blood glucose levels [15]. Two meta-analyses studies assessing how DPP-4 inhibitors influence blood lipid levels showed that they could decrease TG and TC levels but failed to improve HDL level [16, 17]. In addition, these studies imply that DPP-4 inhibitors do not directly act upon blood lipid metabolism, rather DPP-4 inhibitors activate the sympathetic nervous system and thus increase fat mobilization and oxidation [16, 17]. In our study, we did not observe a change in blood lipid level in the sitagliptin treatment group. This could be due to small sample size and short treatment duration. While metformin treatment or sitagliptin treatment alone did not decrease NHDL, combined metformin and sitagliptin treatment significantly decreased NHDL. Further investigation is warranted to elucidate the molecular mechanism of this combination efficacy on decreasing NHDL. Interestingly, a previous report identified that metformin could increase GLP-1 levels, which augmented the effects of DPP-4 inhibitors [18]. Here, we show that combined metformin and sitaglipin treatment is beneficial for T2DM patient in terms of HbA1c, NHDL, and IL-6 levels.

It has been previously demonstrated that pioglitazone treatment leads to an increase of lipoprotein lipase (LPL) expression and the acceleration of triglyceride degradation by activating PPAR-*γ*. Pioglitazone can also partially activate PPAR-*α*, which leads to effect similar to fibrates and an increase of HDL-C [19]. We did not observe an improvement of CCA-IMT in any of the treatment groups, which could be due to the short treatment duration and less severity of the atherosclerosis in the enrolled patient population.

## 5. Conclusion

Taken together, our study demonstrates that metformin, sitagliptin, and pioglitazone all showed safety and efficacy in reducing blood glucose levels, as well as in promoting an anti-inflammatory effect. Our results suggest that combined metformin and sitagliptin treatment may be more beneficial for the improvement of atherosclerosis in patients with T2DM; however, further studies would need to be performed that are extended in duration or recruit a patient population showing signs of increased CCA-IMT prior to treatment to potentially observe an effect directly on development of atherosclerosis.

## Figures and Tables

**Table 1:** Metabolic group parameters of patients at the beginning and at the end of the study.

Parameters	Beginning (*n* = 77)	End (*n* = 71)	*P*
FBG (mmol/L)	7.54 ± 1.77	7.00 ± 1.25	0.04
SBP (mmHg)	131.36 ± 15.25	131.46 ± 14.27	0.97
HbA1c (%)	6.80 ± 0.99	6.46 ± 0.77	0.03
BMI (kg/m^2^)	28.35 ± 2.19	27.91 ± 1.99	0.20
TC (mmol/L)	4.69 ± 0.91	4.51 ± 0.75	0.19
TG (mmol/L)	2.19 ± 1.82	2.03 ± 1.33	0.55
NHDL (mmol/L)	3.75 ± 0.91	3.47 ± 0.77	0.06
HDL (mmol/L)	0.99 ± 0.21	1.03 ± 0.18	0.29
LDL (mmol/L)	2.75 ± 0.78	2.57 ± 0.82	0.21
UA (*μ*mol/L)	336.29 ± 90.55	324.77 ± 92.08	0.44
IMT (mm)	1.22 ± 0.14	1.16 ± 0.14	0.07
Cr (*μ*mol/L)	84.90 ± 14.18	85.96 ± 14.43	0.65
IL-6 (pg/mL)	14.59 ± 5.95	12.63 ± 5.45	0.04
ADPN (ng/mL)	12.18 ± 3.79	13.06 ± 3.80	0.16

**Table 2:** Combined treatment group parameters of patients at the beginning and at the end of the study.

Parameters	Beginning (*n* = 28)	End (*n* = 26)	*P*
FBG (mmol/L)	7.14 ± 1.39	6.38 ± 1.31	0.04
SBP (mmHg)	132.36 ± 15.52	133.35 ± 18.70	0.83
HbA1c (%)	7.32 ± 0.91	6.64 ± 1.08	0.02
BMI (kg/m^2^)	29.53 ± 2.02	28.63 ± 1.51	0.07
TC (mmol/L)	4.50 ± 0.64	4.35 ± 0.57	0.37
TG (mmol/L)	2.23 ± 1.81	2.06 ± 1.66	0.72
NHDL (mmol/L)	3.89 ± 0.85	3.25 ± 1.10	0.03
HDL (mmol/L)	1.01 ± 0.24	1.08 ± 0.28	0.46
LDL (mmol/L)	2.64 ± 0.76	2.36 ± 0.67	0.18
UA (*μ*mol/L)	351.18 ± 87.69	345.35 ± 79.11	0.80
IMT (mm)	1.27 ± 0.12	1.15 ± 0.15	0.05
Cr (*μ*mol/L)	87.29 ± 12.72	90.31 ± 11.65	0.37
IL-6 (pg/mL)	18.70 ± 5.28	15.18 ± 5.18	0.02
ADPN (ng/mL)	11.02 ± 3.40	11.93 ± 3.40	0.33

**Table 3:** Sitagliptin group parameters of patients at the beginning and at the end of the study.

Parameters	Beginning (*n* = 31)	End (*n* = 29)	*P*
FBG (mmol/L)	7.59 ± 1.98	7.03 ± 1.93	0.27
SBP (mmHg)	132.87 ± 16.65	137.66 ± 13.10	0.22
HbA1c (%)	7.14 ± 1.16	6.50 ± 0.70	0.03
BMI (kg/m^2^)	22.02 ± 1.14	21.93 ± 1.24	0.77
TC (mmol/L)	4.81 ± 0.94	4.56 ± 1.02	0.33
TG (mmol/L)	1.43 ± 1.14	1.36 ± 0.92	0.79
NHDL (mmol/L)	3.68 ± 0.90	3.20 ± 1.48	0.29
HDL (mmol/L)	1.00 ± 0.23	1.09 ± 0.25	0.30
LDL (mmol/L)	2.65 ± 0.87	2.53 ± 0.76	0.64
UA (*μ*mol/L)	377.65 ± 81.56	379.00 ± 72.47	0.95
IMT (mm)	1.18 ± 0.16	1.15 ± 0.10	0.59
Cr (*μ*mol/L)	87.35 ± 13.62	86.72 ± 16.82	0.87
IL-6 (pg/mL)	15.82 ± 6.20	12.78 ± 4.30	0.03
ADPN (ng/mL)	11.73 ± 3.67	12.15 ± 3.97	0.67

**Table 4:** Pioglitazone group parameters of patients at the beginning and at the end of the study.

Parameters	Beginning (*n* = 40)	End (*n* = 37)	*P*
FBG (mmol/L)	7.64 ± 1.99	7.14 ± 1.62	0.23
SBP (mmHg)	137.88 ± 16.89	137.44 ± 19.38	0.92
HbA1c (%)	6.94 ± 0.91	6.45 ± 0.70	0.04
BMI (kg/m^2^)	25.94 ± 1.45	26.99 ± 1.87	0.01
TC (mmol/L)	4.61 ± 0.91	4.55 ± 0.79	0.75
TG (mmol/L)	1.88 ± 1.44	1.90 ± 1.44	0.95
NHDL (mmol/L)	3.68 ± 0.92	3.40 ± 1.32	0.37
HDL (mmol/L)	1.01 ± 0.24	1.16 ± 0.23	0.02
LDL (mmol/L)	2.69 ± 0.96	2.52 ± 0.76	0.42
UA (*μ*mol/L)	371.28 ± 73.87	370.76 ± 75.33	0.98
IMT (mm)	1.20 ± 0.14	1.16 ± 0.12	0.26
Cr (*μ*mol/L)	84.48 ± 15.06	82.43 ± 13.49	0.53
IL-6 (pg/mL)	14.32 ± 3.97	12.27 ± 3.91	0.03
ADPN (ng/mL)	11.77 ± 3.36	14.06 ± 4.44	0.01
